# Homœopathic Dilutions

**Published:** 1891-10

**Authors:** 


### Homœopathic Physician,

A MONTHLY JOURNAL OF

HOMŒOPATHIC MATERIA MEDICA AND CLINICAL MEDICINE.

"If our school ever gives up the strict inductive method of Hahnemann, we are lost, and deserve only to be mentioned as a caricature in the history of medicine."—CONSTANTINE HERING.

Vol. XI. OCTOBER, 1891. No. 10.

### EDITORIAL.

Homœopathic Dilutions.—An esteemed correspondent, who is investigating Homœopathy, writes us as follows:

"One thing puzzles me, and in a general question I may ask how is it, when in our food we take many substances that may be used as medicines in your pharmacy (*i. e.*, certain salts and vegetable constituents), that these substances in health are inert in their medicinal actions? * * *

"If a drug like Natrum-muriaticum, for example, which we eat in nearly all our food, has a definite action, how is it that its action is not observed every time we eat salt?"

This question has been asked many times, alike by friends and foes of Homœopathy; the latter, of course, with the intention of baffling the homœopathist and putting him, if possible, in an illogical attitude.

To make this point intelligible to the reader, we must consider some of the physical characteristics of matter.

Matter is made up of atoms. These atoms are at definite distances from each other, even in the hardest, densest substances. This short distance of the atoms apart gives them an orbit, as it were, in which to move. All exhibitions of heat are but the motion of these atoms within their prescribed limits. The same is true of the phenomena of light and electricity.

When a substance is in its crystalline state its individual atoms are comparatively inert. The chemist who would produce desired reactions with such substances knows that he must dissolve them in water, that the distance between the atoms may be so increased and these atoms be far enough removed from the sphere of each other's affinity that they are free to move and produce the desired chemical phenomena. So, too, in the use of any substance as a medicine. The individual atoms must be removed from too close contact with each other, in order that the peculiar influence which they are capable of exerting shall have full opportunity of action. The preparation of the substance for this medicinal action, then, is the same in principle as for its action under chemical conditions. Therefore we triturate, dilute, and succuss.

To make the idea clearer, we may have recourse to a simple illustration.

Suppose we have a cigar-box, furnished with a lid and otherwise in good order. Suppose we fill the box exactly full of marbles and shut down the lid. If, now, we shake the box, no movement of the marbles among themselves takes place. If we remove some of the marbles, and then shake the box, there is a considerable movement of the remainder. Remove all the marbles but a few, and then shake the box, and the freedom of movement among them will be such that they will strike violently against the sides and ends of the box, and may even be made to force the sides out. Yet this could not be done if the box were *full* of marbles.

The box full of marbles may be considered to represent a medicinal substance in its crude state. The box with but a few marbles in it may be considered to represent the same substance after trituration.

Yet another illustration may be given. When lightning passes from a cloud to the earth, the resistance of the atmosphere causes it to dart this way and that in a narrow, zigzag line. This action of lightning may be imitated in the laboratory by an instrument for generating electricity called the Rhumkorff Coil. If such an instrument be made to discharge its electrical current in a glass jar that is closed except for a tube that connects it with an air pump, the electrical current will follow a zigzag line in much the same way as a stroke of lightning. If the air pump be now worked so as to remove a small portion of air from the jar the spark is observed to become broader and more direct. Exhaust the air still more and the spark is no longer a line but spreads out into a purplish light that fills the whole jar. Here we see a parallelism with the marbles in the box.

If now we exhaust all the air from the jar except a few molecules, the electrical current will hurl these particles from one end of the jar to the other with considerable mechanical force. Prof. Crookes has taken advantage of these facts to construct small glass tubes containing small paddle wheels which are made to revolve by the few molecules of air contained in the tubes thus hurled from end to end by the electrical current. Fluorescent crystals are made to glow by the same means. To this condition he has given the name of "radiant matter" or "the fourth condition of matter."

These tubes so prepared are well known among scientific men as "Crookes' Tubes." They are, however, only modifications of the celebrated "Geissler Tubes" and "Plücker Tubes," in which electricity is made to pass through an incomplete vacuum in which are present minute portions of some selected gas or liquid upon whose atoms the electrical current acts.

An attentive consideration of the foregoing explanations will enable us to form some idea of the reason why salt in its crystalline state will not produce any medicinal action to speak of, and yet when triturated or diluted in the manner of all homœopathic preparations will become an active remedy. It also shows the glaring injustice of the derisive description of homœopathic dilutions given by the enemies of Homœopathy when they say that a drop of the tincture is put into a hogshead of water or into the ocean and then a teaspoonful of the mixture is taken. A drop of tincture put into a hogshead of water would not be diffused through the water. It must be put into only a small portion of the menstruum and that must be succussed in order to bring about a separation of the molecules from each other. In this way only will we able to get the medicinal effect.

